# Acute lung injury and post-cardiac arrest syndrome: a narrative review

**DOI:** 10.1186/s40560-024-00745-z

**Published:** 2024-09-03

**Authors:** Yusuke Endo, Tomoaki Aoki, Daniel Jafari, Daniel M. Rolston, Jun Hagiwara, Kanako Ito-Hagiwara, Eriko Nakamura, Cyrus E. Kuschner, Lance B. Becker, Kei Hayashida

**Affiliations:** 1 Laboratory for Critical Care Physiology, Feinstein Institutes for Medical Research, Northwell Health System, Manhasset, NY USA; 2 Department of Emergency Medicine, Donald and Barbara Zucker School of Medicine at Hofstra/Northwell, Hempstead, NY USA

**Keywords:** Post-cardiac arrest syndrome, Post-arrest lung injury, Lung-protective ventilation, Therapeutic hypothermia, Mitochondrial dysfunction, Critical care, Cardiopulmonary resuscitation

## Abstract

**Background:**

Post-cardiac arrest syndrome (PCAS) presents a multifaceted challenge in clinical practice, characterized by severe neurological injury and high mortality rates despite advancements in management strategies. One of the important critical aspects of PCAS is post-arrest lung injury (PALI), which significantly contributes to poor outcomes. PALI arises from a complex interplay of pathophysiological mechanisms, including trauma from chest compressions, pulmonary ischemia–reperfusion (IR) injury, aspiration, and systemic inflammation. Despite its clinical significance, the pathophysiology of PALI remains incompletely understood, necessitating further investigation to optimize therapeutic approaches.

**Methods:**

This review comprehensively examines the existing literature to elucidate the epidemiology, pathophysiology, and therapeutic strategies for PALI. A comprehensive literature search was conducted to identify preclinical and clinical studies investigating PALI. Data from these studies were synthesized to provide a comprehensive overview of PALI and its management.

**Results:**

Epidemiological studies have highlighted the substantial prevalence of PALI in post-cardiac arrest patients, with up to 50% of survivors experiencing acute lung injury. Diagnostic imaging modalities, including chest X-rays, computed tomography, and lung ultrasound, play a crucial role in identifying PALI and assessing its severity. Pathophysiologically, PALI encompasses a spectrum of factors, including chest compression-related trauma, pulmonary IR injury, aspiration, and systemic inflammation, which collectively contribute to lung dysfunction and poor outcomes. Therapeutically, lung-protective ventilation strategies, such as low tidal volume ventilation and optimization of positive end-expiratory pressure, have emerged as cornerstone approaches in the management of PALI. Additionally, therapeutic hypothermia and emerging therapies targeting mitochondrial dysfunction hold promise in mitigating PALI-related morbidity and mortality.

**Conclusion:**

PALI represents a significant clinical challenge in post-cardiac arrest care, necessitating prompt diagnosis and targeted interventions to improve outcomes. Mitochondrial-related therapies are among the novel therapeutic strategies for PALI. Further clinical research is warranted to optimize PALI management and enhance post-cardiac arrest care paradigms.

**Graphical Abstract:**

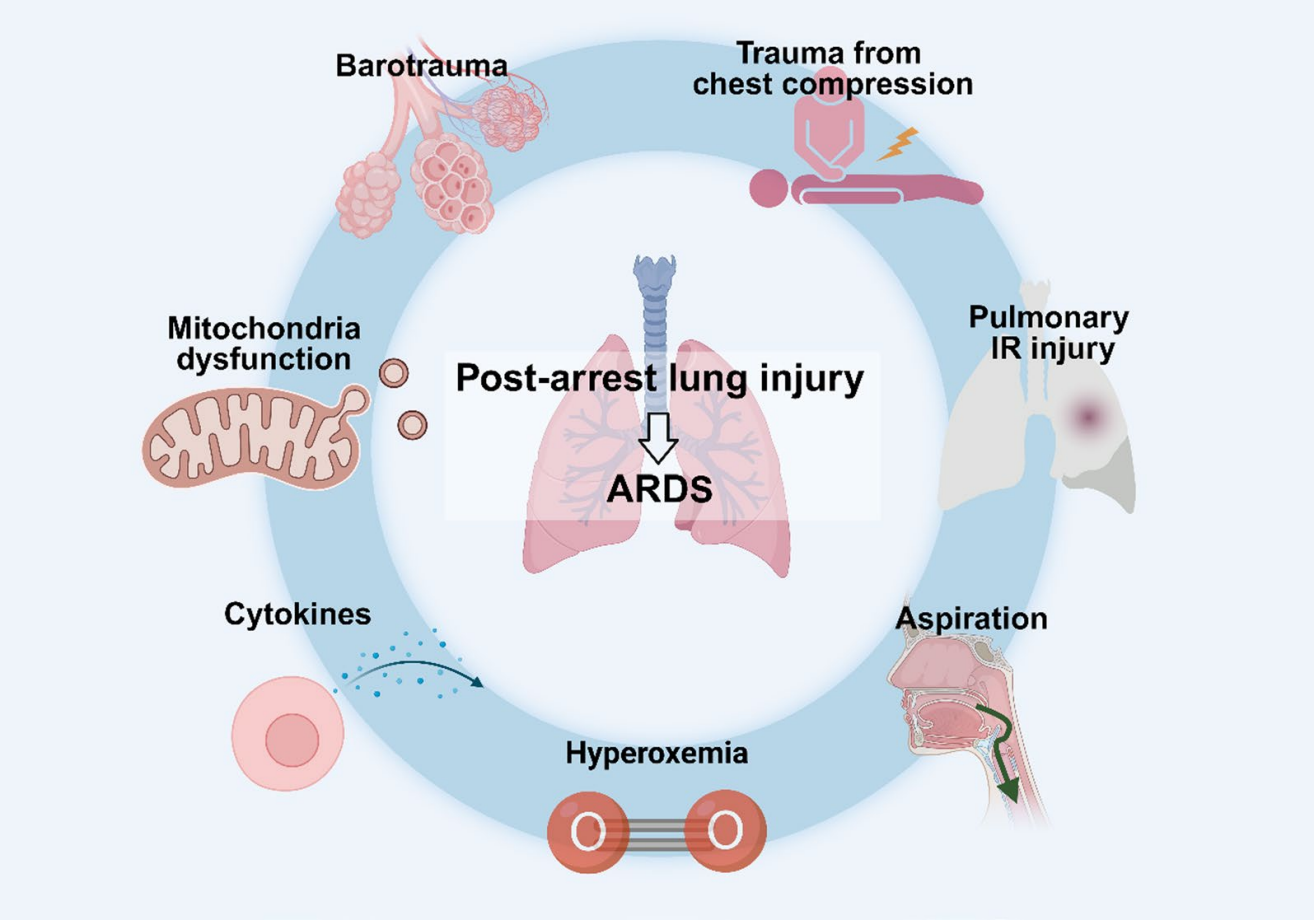

## Introduction

Despite advancements in the management of post-cardiac arrest syndrome (PCAS), a significant proportion of patients with PCAS experiences severe neurological injury and high mortality rates [1–4]. PCAS is characterized by a complex interplay of systemic inflammation, ischemia–reperfusion (IR) injury, and multi-organ dysfunction, all of which contribute to the overall poor prognosis [5–8]. Emerging evidence has identified the lungs as a crucial organ after cardiac arrest (CA) and cardiopulmonary resuscitation (CPR), with lung injuries including acute respiratory distress syndrome (ARDS), significantly impacting patient outcomes [9–14].

The pathogenesis of post-arrest lung injury (PALI) is complex, involving trauma from chest compressions, pulmonary IR injury, aspiration, hyperoxia, and systemic inflammation [9, 11, 12, 15]. The specific underlying mechanisms remain still largely elusive, necessitating further research. This review aims to elucidate the complex pathophysiological landscape of PALI and emphasize the importance of lung-protective strategies in improving patient outcomes post-CA.

## Epidemiology

The incidence and clinical significance of PALI have gained substantial attention in recent years. Emerging evidence have suggested the prevalence of pulmonary complications in the aftermath of CA, particularly following the successful return of spontaneous circulation (ROSC), with up to 50% of CA survivors developing acute lung injury (ALI) [11, 16–19] (Table 1). Clinical studies have identified lung injury rates as high as 79% in patients undergoing CPR, as detected by computed tomography (CT) [16, 17]. The ALI following CPR can manifest as lung edema, thoracic skeletal injuries, and lung IR injury [17, 18]. These findings underscore the substantial burden of lung injury in PCAS, necessitating comprehensive evaluation and management of pulmonary complications.

**Table 1:** Studies reporting the prevalence of post-arrest lung injury in post-cardiac arrest syndrome

Study	Year	Number of patients	%PALI (%)	Modality
Cho et al*.* [16]	2013	44	79.5	CT
Cha et al*.* [17]	2017	91	41.0	CT
Ryu et al. [19]	2019	72	88.9	CT
Jang et al. [18]	2020	43	93.0	CT
Magliocca et al*.* [11]	2021	52	50.0	CT

## Pathophysiology

### Primary causes of PALI

The multifactorial etiologies, which contribute to the complex pathophysiology of PALI, include trauma induced by chest compressions, barotrauma resulting from mechanical ventilation, aspiration during and after CA/CPR, and IR injury within the pulmonary system post-CA [15].

Traumatic lung injury, which manifests as pulmonary contusion induced by chest compression and barotrauma, is frequently observed with pathologic features such as pulmonary hemorrhage, pulmonary edema, and atelectasis secondary to physical disruption of alveolar capillary membranes [14, 20]. The incidence of chest compression-related pulmonary contusions ranges from 41 to 100%, with bilateral lung contusions seen in most of CA patients [16–19].

Aspiration is frequently observed during CA/CPR, with a high incidence of 20–65% [21–24]. In particular, in patients with OHCA, aspiration of gastric contents has been reported to occur in approximately 30% of patients during or after CPR [25, 26]. Aspiration of acidic gastric fluid or oropharyngeal secretions can result in increased alveolar-capillary permeability, further increasing inflammation and exacerbating lung injury [24]. Complications of aspiration in patients with CA can lead to respiratory failure. One study reported that aspiration was associated with a significantly lower 30-day survival rate compared to CA due to other causes of respiratory failure [27]. Although it is often difficult to determine whether aspiration occurred before, during, or after CPR, a higher level of care needs to be considered to prevent aspiration and the subsequent severity of PALI.

Pulmonary IR injury is a complex pathophysiological process that significantly contributes to morbidity and mortality in a wide range of clinical scenarios, including PCAS, lung transplantation, cardiopulmonary bypass, and ARDS. This condition is marked by a pronounced increase in alveolar-capillary permeability, a hallmark of the disrupted pulmonary barrier function [28]. At the cellular level, IR injury in the lung is characterized by the induction of apoptosis and the robust production of reactive oxygen species (ROS), initiating a cascade of inflammatory responses. Central to this inflammatory milieu is the upregulation of a spectrum of cytokines, including tumor necrosis factor-alpha (TNF-α), interferon-gamma (IFN-γ), and a series of interleukins such as interleukin (IL)-8, IL-10, IL-12, and IL-18 [29, 30]. These mediators play pivotal roles in orchestrating the local and systemic inflammatory response, exacerbating tissue damage during the critical phases of circulatory disruption and subsequent reperfusion. Cell apoptosis in the lung after IR could be more detrimental to the lung than cell necrosis and associated inflammation [31]. Moreover, recent studies have shown that mitochondrial DNA (mtDNA) has been associated with the development of ARDS [32–36]. Elevated circulating levels of mtDNA are found in patients with ALI, which correlates with poor outcomes [36, 37], suggesting plasma mtDNA released from damaged mitochondria as damage-associated molecular patterns (DAMPs) may induce lung injury.

### ARDS post-CA

ARDS frequently follows both out-of-hospital cardiac arrest (OHCA) [38] and in-hospital cardiac arrest (IHCA) [39], with PALI presenting as a spectrum of pulmonary damage. The multifaceted nature of PALI, compounded by systemic IR injury and inflammatory response, can exacerbate poor outcomes, with high oxygen concentrations during CPR, pulmonary contusions, and aspiration further increasing the risk of ARDS through oxidative stress mechanisms [40–42]. A retrospective analysis of OHCA patients by Johnson et al*.* reported a 48% incidence of ARDS among mechanically ventilated OHCA patients, with an average *P*/*F* ratio of 155 mmHg, corresponding to moderate ARDS. Shih et al. reported that approximately three-quarters of IHCA patients experienced ARDS post-ROSC [39]. In an unadjusted analysis, the presence of ARDS was linked to fewer alive-and-ventilator-free days across 28 days with a median of 1 day as compared to 18 days in patients without ARDS, though this association did not achieve statistical significance upon multivariate analysis [39]. These findings indicate that ARDS is a frequent complication post-CA, suggesting an important area for future therapeutic strategies to improve outcomes post-CA. Given its prevalence and impact, ARDS management in PCAS patients should align with established ARDS protocols to mitigate the risks of prolonged ventilation and increased mortality.

### Lung compliance post-CA

Previous research has demonstrated that pulmonary dysfunction following CA is associated with impaired gas exchange, increased pulmonary edema, and the release of pro-inflammatory cytokines [43]. These factors can further contribute to reduced lung compliance and exacerbate lung injury. Additionally, the utilization of mechanical ventilation, almost always required for the management of PCAS, can impact lung compliance, and potentially contribute to lung injury if not diligently controlled [44].

Respiratory system compliance, consisting of pulmonary and chest wall compliance, refers to the lung capacity to expand and adapt to volume changes during the respiratory cycle. Pulmonary components are influenced by various factors, including lung tissue elasticity and the presence of pathologic conditions. The chest wall compliance is often affected by intrinsic respiratory muscle tone, and certain pathological conditions such as deformities of the chest wall from traumatic rib fractures or burns. Following CPR, decreased lung compliance may be a result of factors such as IR injury, systemic inflammation, and ventilation-associated lung injury (VALI). Chest wall compliance may be affected by chest wall trauma from CPR [45, 46]. A study assessing lung compliance in patients with PCAS reported a mean lung compliance of 0.051 ± 0.011 L/cm H_2_O [46], with a notable decrease at lower lung volumes suggesting the risk of alveolar collapse. The duration of CPR further exacerbates this decline in compliance. Furthermore, decreased lung compliance, a prominent feature of ARDS, may explain higher risk of lung injury following CA. This was demonstrated in a study of PCAS patients who developed ARDS [47]. This relationship underscores the need for vigilant respiratory management aimed at preserving lung function and improving survival outcomes with intact neurological function.

### Pulmonary edema and mechanical chest compression

Pulmonary edema following resuscitation is typically transient and considered hydrostatic in nature. However, the exact mechanisms underlying this condition remain to be definitively determined. Potential contributing factors include decreased left ventricular contractility, ineffective chest compressions that fail to adequately propel blood forward, and the reverse flow of blood into the lungs. This backward movement of the blood can cause elevated left ventricular filling pressures and left atrial pressures, further contributing to pulmonary edema during and immediately after CA, while the heart is still stunned.

Mechanical chest compression (MCC) during CPR is associated with an increased incidence of pulmonary edema compared to manual compressions [11]. The pulmonary edema which is characterized by increased lung fluid volume and weight, along with reduced oxygenation and respiratory system compliance, underscores the physiological impact of MCC resuscitation on lung condition [11]. Magliocca et al*.* demonstrated that MCC notably exacerbates CPR-induced lung edema, a finding consistent across animal models and OHCA patients [11]. However, no worsening has been observed in survival to discharge or length of ICU stay due to the MCC effect. The hemodynamic effects of piston-based devices, while improving blood flow, may also exacerbate vascular congestion by the pressure aspiration effect, hinting at a complex interplay of factors leading to transient pulmonary edema [48].

## Assessment and diagnosis

Diagnostic imaging plays a crucial role in evaluating the causes and complications after CA/CPR. Imaging techniques, such as chest x-rays, CT, and lung ultrasound, are instrumental in identifying common lung injury manifestations, such as ground-glass opacities predominantly located in the posterior segments of the lungs [16]. Lung injuries resulting from chest compressions during CPR can lead to pulmonary hemorrhage and edema. These conditions stem from the disruption of alveolar-capillary membranes, pulmonary IR injury, and the aspiration of gastric and oropharyngeal secretions as described above. The differentiation between aspiration pneumonia and lung injury through imaging can be challenging due to overlapping patterns in dependent lung areas [16].

Chest x-rays can be used to identify chest complications after CA/CPR, including rib and sternal fractures, pericardial effusions, and lung injury [49]. A study involving chest x-rays of 44 patients post-CA/CPR revealed increased opacities in 63.6% of the cases, underscoring the prevalence of lung injuries [16]. However, CT scans within the same study group showed a higher detection rate of lung injuries at 79.5%, including in patients with normal chest x-rays, highlighting CT’s superior diagnostic capability [16].

Quantitative CT evaluation offers a detailed assessment of lung injury severity after successful CPR [14]. A study using a porcine ventricular fibrillation model revealed ALI on CT, such as intense parenchymal and ground-glass opacifications, which are indicative of pulmonary edema and alveolar damage, respectively [14]. Further, these changes were more sensitive than the oxygenation index using blood gas analysis, indicating CT’s enhanced sensitivity over traditional blood gas analysis. The utility of CT in detecting pulmonary edema post-resuscitation has been validated in both animal studies and human cases, emphasizing the need for early CT examination for comprehensive lung injury assessment post-CA/CPR [11]. Clinically, CT must be considered standard of care when feasible in PCAS.

Lung ultrasound emerges as a rapid and non-invasive tool for bedside lung injury assessment and monitoring lung injury post-CA [50, 51]. We et al. reported that the lung ultrasound score (LUS), calculated from four findings; (1) presence of lung sliding with A lines or fewer than two isolated B lines, (2) well-defined B lines, (3) multiple coalescent B lines, (4) presence of tissue pattern characterized by dynamic air bronchograms, was useful in assessing lung status and degree of lung injury in a porcine CA model [10].

The extravascular lung water (EVLW) and pulmonary vascular permeability index (PVPI) by the pulse index continuous cardiac output (PiCCO) technology using transpulmonary thermodilution are useful markers of severity of lung injury [52]. In ARDS patients, a strong correlation exists between LUS, EVLW, and PVPI, with early measurements serving as good prognostic indicators [53, 54]. Given the significant correlation of these markers in both ARDS and post-CA contexts, PiCCO technology could aid in detecting lung injuries in PCAS patients, albeit with considerations for its cost, invasiveness, and complexity [10].

## Potential therapeutic strategies for PALI

### Tidal volume management

Following CA/CPR, up to 50% of resuscitated patients develop lung injury that meets the criteria for ARDS during their intensive care unit (ICU) stay, highlighting the need for lung-protective ventilation strategies in patients with PCAS [38]. A meta-analysis of critically ill non-CA patients without ARDS reported that low tidal volume (*V*_T_) was associated with lower lung infection, atelectasis, and mortality [55]. Despite limited data on ventilator management for PCAS, current practices often involve higher *V*_T_ and driving pressures (Δ*P*), which may exacerbate ventilator-induced lung injury (VILI) [56]. In a study using a two-center retrospective cohort of OHCA patients, Beitler et al. showed that lower *V*_T_ [< 8 ml/kg predicted body weight (PBW)] was associated with improved functional outcomes [57]. Furthermore, a preplanned sub-analysis of the Target Temperature Management (TTM)-2 trial by Robba et al. has demonstrated that mechanical output, Δ*P*, and ventilation rate, as well as positive end-expiratory pressure (PEEP), respiratory plateau pressure (*P*_PLAT_), and single *V*_T_, were independently associated with 6-month mortality in the post-resuscitation respiratory setting [58]. European guidelines advocate for *V*_T_ of 6–8 ml/kg PBW in brain-injured and post-CA patients, although evidence in the post-CA context is sparse [59]. These findings underscore the critical need for lung-protective strategies with lower *V*_T_ (6–8 ml/kg PBW) to prevent VILI, advocating for more careful and frequent adjustments of ventilator settings in the PCAS care to avoid *V*_T_ higher than 8 ml/kg PBW. To meet the ventilatory needs of the patient, respiratory rate should be adjusted to allow for a higher minute ventilation to avoid hypercarbic respiratory failure and potential worsening brain injury [60]. However, it must be emphasized that given frequently hypermetabolic state of PCAS, very high respiratory rates may lead to inappropriately high airway pressures which the clinician should be vigilantly monitoring. Lastly, it must be noted that unless the patient is deeply sedated or paralyzed, the respiratory rate may be centrally driven to be high, irrespective of the respiratory rate set by the ventilator.

### Managing hypercapnia

Along with the proper management of *V*_T_, the impacts of hypercapnia on cerebral blood flow and intracranial pressure (ICP) are critical considerations in post-CA care. Studies reported that mild hypercapnia improved cerebral oxygenation but did not increase ICP or cerebral edema, suggesting potential therapeutic benefits in the acute phase following resuscitation [61, 62]. However, a recent randomized controlled trial (RCT) challenged this notion by demonstrating that, in comatose patients resuscitated after OHCA, targeted mild hypercapnia did not result in superior neurological outcomes at 6 months compared to targeted normocapnia [63]. Despite this, the absence of consistent evidence regarding the optimal role of hypercapnia post-CA has not deterred the clinical practice of permitting mild hypercapnia as an integral component of lung-protective ventilation strategies. This approach is primarily aimed at reducing the risk of VILI while potentially aiding cerebral perfusion. As such, PaCO_2_ levels are often maintained within a target range of 35 to 50 mmHg, striking a balance between ensuring sufficient oxygenation and mitigating the risk of negative cerebral consequences [64]. This nuanced approach underscores the complexity of managing ventilation in post-CA patients and highlights the need for ongoing research to refine these strategies for optimal patient outcomes.

### Optimizing PEEP, plateau pressure, and driving pressure

PEEP is a crucial aspect of mechanical ventilation strategy, especially in the management of PCAS, yet research specifically addressing its optimal use in this patient population remains limited. The application of PEEP for patients with PCAS requires careful consideration, given its potential to influence both pulmonary and cerebral hemodynamics significantly. On one hand, high levels of PEEP can pose risks, such as diminished tissue oxygen delivery resulting from diminished venous return, circulatory impairment and elevated ICP. On the other hand, setting PEEP too low or employing zero PEEP might increase the likelihood of atelectasis and subsequent lung injury, complicating the patient’s recovery process [65, 66]. Sutherasan et al. have shown that higher *V*_T_, higher *P*_PLAT_, and lower PEEP in the first 24 h after ICU admission were associated with an increased risk of developing ARDS or pneumonia in patients initially without lung injury [44]. This emphasizes the need for careful ventilatory settings to prevent secondary lung complications. Thus, setting PEEP at ≥ 5 cmH_2_O is advisable to prevent atelectasis and protect against PALI, while also considering risks such as dynamic lung hyperinflation, circulatory depression, and brain damage [64, 67].

Plateau pressure (*P*_PLAT_) is a crucial measure in mechanical ventilation, representing the pressure in the airways at the end of inspiration without airflow, thus reflecting mean alveolar pressure without being influenced by airway resistance. Keeping *P*_PLAT_ < 20 cmH_2_O is recommended to minimize mortality risk, especially in patients without ARDS. This is supported by a study in PCAS cohorts, where maintaining *P*_PLAT_ < 20 cmH_2_O has been linked to better outcomes and reduced risk of barotrauma, a common complication after resuscitation [58]. *P*_PLAT_ < 20 cmH_2_O may be difficult in post-cardiac arrest patients requiring higher PEEP levels.

Driving pressure (Δ*P*) is reflecting the stress exerted during the lung expansion, calculated as the difference between *P*_PLAT_ and PEEP. A sub-analysis of the TTM1 trial reported a median Δ*P* of 14.7 cmH_2_O in patients with PCAS, with findings indicating that Δ*P* was an independent factor of higher mortality and adverse neurological outcomes [58]. However, Δ*P* does not consider respiratory rate, which itself can contribute to VALI, which is part of PALI [68]. Lowering Δ*P* might lead to increase PaCO_2_ due to reduced *V*_T_, necessitating a higher respiratory rate to maintain constant PaCO_2_ levels. For this reason, an equation incorporating both Δ*P* and respiratory rate (4 × Δ*P* + respiratory rate) was explored in patients with ARDS and found to strongly correlate with mortality [69]. This metric was also applied by Robba et al. in a cohort of post-CA patients, revealing a robust association with poor neurological outcomes and increased mortality [58]. Currently, it is suggested to keep the Δ*P* < 13 cmH_2_O by adjusting the *V*_T_ and PEEP level according to the patient’s clinical picture, aiming to optimize lung-protective ventilation strategies [64].

### Extra corporeal membrane oxygenation

The extra corporeal membrane oxygenation (ECMO) has been used an extension of CPR (extracorporeal CPR: ECPR) in recent years [70–72]. In addition to potentially improved survival rates with favorable neurological outcomes, ECMO is a valuable modality to minimize the risk of PALI, pulmonary congestion, and frequently exacerbated ventilatory support, which may interfere with lung protective ventilation discussed above. However, it must be noted that ECMO may exacerbate pulmonary congestion through aortic retrograde flow resulting in increased left ventricular afterload [73]. This can be mitigated by running ECMO at the lowest possible flow wares, implementing a percutaneous micro axial pump, or veno-arterial-venous ECMO configuration [74]. Despite the EOLIA trial’s controversial results, in the case of the development of ARDS, ECMO could be deployed in either veno-venous or veno-arterial configuration to allow for ventilation and oxygenation while the lungs are being rested [75].

### Therapeutic hypothermia

Therapeutic hypothermia, inclusive of TTM, is the primary neuroprotective approach post-CA [76–78]. The efficacy of therapeutic hypothermia for lung injury, including ARDS, has been reported in both animal and human studies. In injury model such as pulmonary IR injury, pulmonary hypertension, VILI, smoking-induced injury, endotoxemia model, and hemorrhagic shock, therapeutic hypothermia has been reported to suppress lung injury by modulating inducible nitric oxide synthase (iNOS) production, endothelial nitric oxide synthase (eNOS) production, neutrophil activation, or adhesion molecule expression [79–86]. An animal study using a rabbit model of ALI has shown that mild therapeutic hypothermia with external cooling reduced lung inflammation and damage and improved oxygenation, likely by lowering levels of inflammatory cytokines, such as TNF-α, IL-6, and IL-8 [87]. In addition, in an experiment using pig models of oleic acid-induced ARDS, authors reported that the intervention of hypothermia at 32 °C reduced the pathological lung damage and improved lung mechanics [88]. A retrospective study of 58 patients with ARDS who received muscle relaxation and a pilot feasibility study of 8 patients with severe ARDS with *P*/*F* ratio < 150 showed that hypothermia (34–36 °C for 48 h) reduced in-hospital mortality (75% vs 53.4%, *p* = 0.26) and increased ventilator-free days, while not statistically significant [89]. Conversely, therapeutic hypothermia is associated with an increased risk of pneumonia, which could worsen PALI [90].

Wu et al. investigated the impact of therapeutic hypothermia on PALI using a swine model of CA [10]. In their study, hypothermic animals were cooled to 33 °C for 24 h post-resuscitation, then gradually rewarmed at 1 °C/h for 5 h, while normothermic animals were kept at 37–38 °C. Despite both groups exhibiting PALI, the hypothermia group showed significant improvements in ELWI, PVPI, and *P*/*F* ratio, indicating reduced lung injury. Although derived from limited animal research, these findings suggest therapeutic hypothermia could potentially be a viable treatment for PALI.

The Hypothermia After Cardiac Arrest (HACA) study [77] and a study by Bernard et al. [76] reported improved neurological prognosis and reduced mortality in patients with ventricular tachycardia or ventricular fibrillation who underwent therapeutic hypothermia, cooled to 32–34 °C, compared to those who were not cooled. These seminal studies have significantly influenced clinical practices in the management of post-CA patients. However, the TTM trial [78] found that hypothermia at 33 °C did not improve mortality or neurological outcomes in patients following OHCA when compared with normothermia at 37 °C. Subsequently, the HYPERION Trial reported a significant improvement in survival and neurological outcomes at 34 °C for patients with pulseless electrical activity or asystole [91]. Yet, the TTM2 Trial indicated no difference in neurological outcomes or mortality between temperatures of 33 °C for 24 h and 36 °C for 24 h [92], suggesting that the effectiveness of hypothermia in PCAS patients remains controversial. Notably, none of these RCTs provides clear evidence regarding the impact of hypothermia on lung function.

### Factors associated with mitochondrial pathophysiology

Mitochondria plays an integral role in the development and onset of ALI. DAMPs are a collective term for numerous endogenous risk molecules present in the nucleus, mitochondria, or cytoplasm [93, 94], and mtDNA, as mitochondrial DAMPs, has been implicated in sepsis-induced increases lung endothelial cell permeability in ALI [95]. The presence of large amounts of ROS can upset the balance between mitochondrial dysfunction and mitosis, accelerating sepsis progression and indirectly causing ALI [96]. Thus, factors related to impaired mitochondrial pathophysiology may be potential therapeutic targets for PALI in patients with PCAS [97]. Mitochondria-targeted antioxidants can protect against ventilator-induced mitochondrial dysfunction and oxidative stress, suggesting improved outcomes for ALI treated by ventilators [98]. Furthermore, mitochondrial transplantation, as an emerging technology to replace damaged mitochondria with exogenous healthy mitochondria [99–101], can significantly improve lung status and reduce lung tissue damage caused by ALI [102]. Pang et al. showed that in an endotoxin-induced ALI rat model, allogeneic mitochondria administered via the jugular vein accumulated in the lungs, protecting the endothelium of alveolar capillary arrays, and improving gas exchange in the acute phase [103]. Moreover, Hayashida et al. showed that intravenous administration of allogeneic mitochondria immediately after ROSC improves the lung wet/dry ratio after resuscitation in a rat model of asphyxial CA [104]. However, since there is currently no clinically available bedside surrogate marker for mitochondrial dysfunction, further translational research is warranted to develop real-time measurement techniques for assessing mitochondrial damage.

### Other therapeutic approaches

A Rho kinase (ROCK) is a type of protein kinase that plays a crucial role in cellular processes such as cell contraction, adhesion, motility, and transcriptional regulation. It is activated by the small GTPase RhoA and regulates the phosphorylation of myosin light chain, which affects the remodeling of the cell cytoskeleton. The Rho/ROCK signaling pathway also influences the expression of vascular endothelial-cadherin, which maintains endothelial junction stability, and intercellular adhesive molecule-1, which regulates leukocyte adhesion and transmigration. ROCK inhibition has been found to be effective in reducing damage caused by IR injury and preventing neutrophil recruitment and edema formation in ALI. Fasudil, a Rho kinase inhibitor, is currently used clinically to treat cerebral vasospasms and has shown promising safety profiles in various clinical trials for conditions such as angina, systemic and pulmonary hypertension, stroke, and heart failure. One study demonstrated that pretreatment with fasudil, a medication, can reduce lung injury caused by CA in rats [105]. Fasudil showed protective effects by decreasing lung edema, oxidative stress, and inflammation. These effects are believed to be mediated through the inhibition of the Rho/ROCK signaling pathway, which is known to be involved in lung IR injury.

Alda-1 is a compound that activates aldehyde dehydrogenase 2 (ALDH2), an enzyme involved in the removal of toxic aldehydic products. The specific agonist of ALDH2, Alda-1, has been shown to protect the lung against different stimuli in various experimental settings, such as acrolein-, hypoxia-, sepsis-, heatstroke-, severe hemorrhagic shock-induced lung injury, regional lung IR injury, and global IR injury [106–111]. One study aimed to investigate whether Alda-1 treatment could alleviate lung injury after CA/CPR in a swine model [112]. They found that Alda-1 improved lung function and reduced cell death through the inhibition of apoptosis and ferroptosis, suggesting its potential as a therapeutic approach for lung protection and enhance cell survival in PALI.

## Future directions

**Fig. 1:**
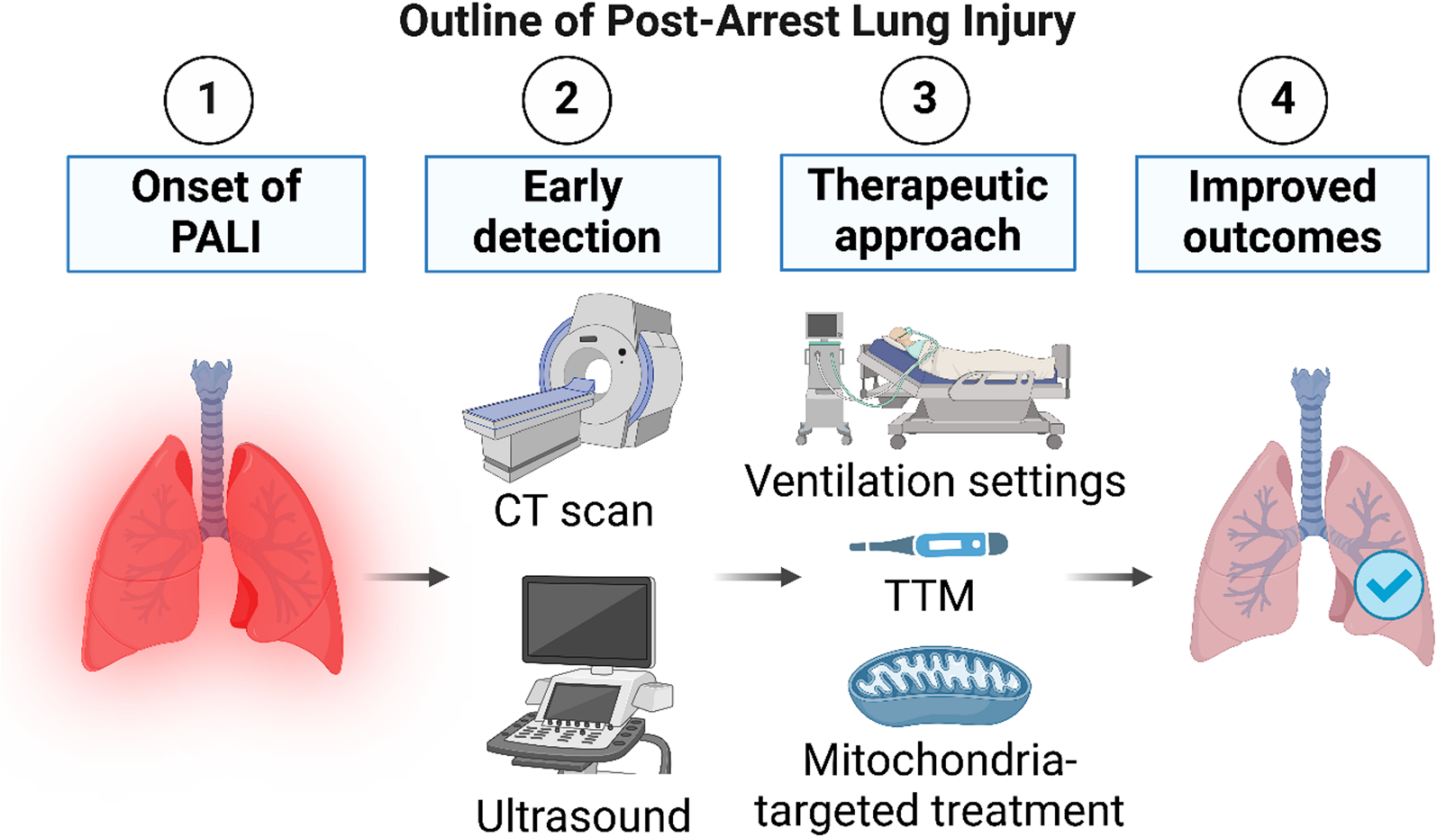
A clinical outline of post-arrest lung injury

An overview of PALI outline in our knowledge to date is summarized in Fig. 1. PALI is thought to be caused by a complex combination of factors associated with CA. The impact of each factor probably varies from the patient’s background, including underlying disease and duration of CA. On the other hand, the incidence and pathogenesis of PALI are gradually becoming clearer with the widespread use of rapid post-arrest CT scans and lung ultrasound. Although the severity of PALI varies, it has been reported that the presence of PALI affects the prognosis of patients after CA, and thus the treatment of PALI and prevention methods are expected to be studied [38, 47, 113–115].

ARDS presents primarily as hypoxic respiratory failure, especially in the early acute phase. Systemic and brain tissue hypoxia are associated with worse outcomes in PCAS, and it has been reported that patients who develop ARDS after OHCA are less likely to recover neurologically and be discharged [38]. The association between ventilation at low tidal volume and improved outcomes after OHCA has been demonstrated, and palliation or risk reduction of ARDS may be an important treatment strategy for improving outcomes, including neurologically intact survival after CA [57]. Currently, the treatment of PALI is similar to that of ALI and ARDS, with appropriate ventilator settings in post-ROSC respiratory management being critical. In addition, therapeutic hypothermia has been reported to reduce lung injury in animal models as well as in clinical cases. Particularly, in PCAS patients with severe PALI, the reduction of lung injury by TTM may be more important than these side effects, so it is necessary to investigate the settings of TTM and patient criteria for selecting TTM for the treatment of PALI.

In PCAS, mitochondrial dysfunction manifests distinctly across different organs, influenced by their specific metabolic demands and stress responses [116]. In the lungs, increased oxidative stress and impaired mitochondrial respiration lead to the release of mtDNA, which acts as a DAMP, eliciting inflammatory responses that contribute to ALI. In the brain, mitochondrial dysfunction primarily involves extensive oxidative damage and impaired ATP production, leading to neuronal cell death and neurodegeneration. Cardiac mitochondria experience disrupted electron transport chain function, reduced ATP synthesis, and increased ROS production, resulting in oxidative damage and enhanced apoptosis. Renal mitochondrial alterations include impaired biogenesis and function, elevated oxidative stress, and altered dynamics affecting cellular homeostasis and survival.

The interaction of organs in PCAS is critical due to the interdependence of their functions and the systemic nature of the response to IR injury. Mitochondrial dysfunction in one organ can exacerbate dysfunction in others, creating a vicious cycle of damage. For example, acute lung injury (ALI) increases systemic inflammation and oxidative stress, worsening outcomes in the brain, heart, and kidneys. Understanding these interactions is essential for developing comprehensive treatment strategies that address multi-organ dysfunction in PCAS.

Improvement of mitochondrial function in patients with lung injury is thought to have the potential to improve prognosis as well as lung injury. In particular, mitochondrial transplantation could be a promising therapy to improve lung injury post-CA. However, while the use of animal models has shown that mitochondrial transplantation can accumulate in the injured lungs and reduce lung damage, there are a number of issues that need to be addressed before it can be applied clinically. These include the source of the mitochondria to be transplanted, the timing of the transplant, the indication, the dosage, and the frequency of mitochondrial transplantation. It is essential to develop translational studies to apply mitochondrial transplantation to clinical practice in particular, and it will be necessary to set conditions for clinical application based on these data.

## Conclusions

PALI is a pathological condition that has a high overall incidence as a complication and affects the prognosis of patients after CA/CPR. Rapid detection of PALI is essential to ensure prompt etiologic therapy, and the use of diagnostic tools is mandatory. Early quantitative CT evaluation is important to improve the accuracy of clinical diagnosis, and the usefulness of LUS has been reported as a bedside assessment of lung injury. Supportive care for patients with PALI should be based on the need to maintain adequate oxygen and ventilator settings while reducing the potential for lung injury due to VILI or other causes. The clinical translational research on pharmacological approaches, including mitochondria-targeted drugs, remains extremely limited. Further research is needed to elucidate which patients benefit from therapeutic hypothermia.

## Data Availability

Not applicable.

## Abbreviations

ALDH: Aldehyde dehydrogenase 2
ALI: Acute lung injury
ARDS: Acute respiratory distress syndrome
CA: Cardiac arrest
CPR: Cardiopulmonary resuscitation
CT: Computed tomography
DAMPs: Damage-associated molecular patterns
ECMO: Extra corporeal membrane oxygenation
EVLW: Extravascular lung water
ICP: Intracranial pressure
ICU: Intensive care unit
IFN-γ: Interferon-gamma
IL: Interleukin
IR: Ishcemia-reperfusion
LUS: Lung ultrasound score
MCC: Mechanical chest compression
mtDNA: Mitochondrial DNA
OHCA: Out-of-hospital cardiac arrest
PALI: Post-arrest lung injury
PCAS: Post-cardiac arrest syndrome
PEEP: Positive end-expiratory pressure
PiCCO: Pulse-induced contour cardiac output
*P*_PLAT_: Plateu pressure
PVPI: Pulmonary vascular permeability index
RCT: Randomized controlled trial
ROCK: Rho kinase
ROS: Reactive oxygen species
ROSC: Return of spontaneous circulation
TNF-α: Tumor necrosis factor-alpha
TTM: Target temperature management
VALI: Ventilator-associated lung injury
VILI: Ventilator-induced lung injury
Δ*P*: Driving pressure
